# Respiratory Physiology and the Impact of Different Modes of Ventilation on the Photoplethysmographic Waveform

**DOI:** 10.3390/s120202236

**Published:** 2012-02-16

**Authors:** Aymen A. Alian, Kirk H. Shelley

**Affiliations:** Department of Anesthesiology, Yale University School of Medicine, 333 Cedar Street, P.O. Box 208051, New Haven, CT 06520, USA; E-Mail: aymen.alian@yale.edu

**Keywords:** clinical monitoring, photoplethysmographic waveform, respiratory physiology, pulse oximeter waveform

## Abstract

The photoplethysmographic waveform sits at the core of the most used, and arguably the most important, clinical monitor, the pulse oximeter. Interestingly, the pulse oximeter was discovered while examining an artifact during the development of a noninvasive cardiac output monitor. This article will explore the response of the pulse oximeter waveform to various modes of ventilation. Modern digital signal processing is allowing for a re-examination of this ubiquitous signal. The effect of ventilation on the photoplethysmographic waveform has long been thought of as a source of artifact. The primary goal of this article is to improve the understanding of the underlying physiology responsible for the observed phenomena, thereby encouraging the utilization of this understanding to develop new methods of patient monitoring. The reader will be presented with a review of respiratory physiology followed by numerous examples of the impact of ventilation on the photoplethysmographic waveform.

## 1. Introduction

Assessment of fluid responsiveness is an important issue in the fluid management of critically ill patients. A fluid challenge in patients with borderline cardiac reserve may result in an overt pulmonary edema, necessitating ventilatory support [1,2]. Static measures such as central venous pressure or the pulmonary artery wedge pressure, if not extremely low, are not useful for assessment of fluid responsiveness [3–5], while dynamic measures such as pulse pressure variation (PPV), systolic pressure variation (SPV) and stroke volume variation (SVV) predict fluid responsiveness well during mechanical ventilation. These variations are caused by tidal changes in the intrathoracic pressure induced by positive pressure ventilation [6–9]. Thus, dynamic preload variables were considered to be important in guiding fluid and catecholamine therapy in critically ill patients [10,11].

During spontaneous ventilation, dynamic parameters like pulse pressure variation (PPV) and systolic pressure variation (SPV) are usually small. Inspiratory and/or expiratory threshold resistors were used in spontaneously breathing pigs as an effort to magnify SPV and PPV during hypovolemia [12].

In the following article the authors will discuss briefly the physiologic impact of mechanical ventilation, spontaneous breathing and incentive spirometry on right and left ventricular preload and afterload. We will then examine how these effect they might appear on photoplethysmographic (PPG) waveforms. Different examples of different modes of ventilation and their impact on plethysmographic waveforms will be given.

For lung volume to expand, transpulmonary pressure (*i.e.*, alveolar pressure-pleural pressure (Ppl)) must increase. This is accomplished by an increase in alveolar pressure during positive pressure inspiration or by a decrease in Ppl during spontaneous inspiration. Because of their anatomic position in the closed thoracic cavity, the heart and lungs interact during each ventilation cycle.

## 2. During Controlled Mechanical Ventilation

### 2.1. Right Heart Effects of Positive-Pressure Ventilation

**Preload** During inspiration, the positive intrathoracic pressure compresses the compliant vena cava and increases resistance to venous flow and results in reductions in venous return [13].

**Afterload** Right ventricular afterload indicators, such as pulmonary vascular resistance and right ventricular output impedance, are augmented during the mechanical ventilation inspiratory cycle as well as spontaneous inspiration. The vascular resistance of alveolar vessels increases as alveoli expands with gas, and vessel compression occurs as alveoli inflate; thus, tidal volume is the primary determinant of right ventricular afterload [14–16]. Augmentation in right ventricular afterload necessitates the right ventricle generate greater pressure to eject blood into the pulmonary artery. Accordingly, right ventricular work and myocardial oxygen demands are increased. Individuals who are unable to sufficiently increase contractile force in response to increased right ventricular afterload have a subsequent reduction in right ventricular stroke volume and ejection fraction. The resultant increase in end-diastolic volume dilates the right ventricle. Ventricular dilation will alter the pressure gradient for coronary artery blood flow, increase ventricular wall tension, and shift the interventricular septum toward the left ventricle [17].

### 2.2. Left Heart Effects of Positive-Pressure Ventilation

**Preload** The reduced right ventricular venous return subsequently decreases left ventricular preload, stroke volume and cardiac output and the increased right ventricular afterload and right ventricular dilation with shift of the interventricular septum. Hyperinflated lungs directly compress the heart, reduce cardiac compliance, and lessen ventricular filling and end diastolic volumes [17,18]. Left ventricular preload may also be influenced by increased pericardial pressure [19].

**Afterload** Positive pressure mechanical ventilation also reduces left ventricular afterload or transmural pressure on inspiration and throughout the ventilatory cycle with the application of PEEP or CPAP [18,20].Transmural pressure is the pressure inside the ventricular chamber minus pressure outside the ventricle (intrathoracic pressure). Left ventricular transmural pressure is an indication of the pressure the ventricle must overcome to eject blood into the aorta. Positive intrathoracic pressure actually unloads the left ventricle by reducing transmural pressure. Consequently, the left ventricle is able to eject a greater stroke volume of blood with less pressure generation. Thus, myocardial oxygen demand is reduced and cardiac output improves.

## 3. During Spontaneous Breathing

Spontaneous inspiration was associated with a simultaneous fall in arterial pressure (Pa), pulmonary artery pressure (PAP) and left atrial pressure (LAP). The pleural pressure (Ppl) fell more than did chamber pressures which resulted in an increased transmural LAP, PAP, RAP (right atrial pressure), and aortic transmural pressure increased. Systemic venous return augmented as a result of increased transmural RAP and the LV afterload increased as the transmural aortic pressure (Pa-Ppl) increased. Both of these factors may have contributed to the increase in left atrial filling pressure (LAP-Ppl).

## 4. During Spontaneous Breathing Using Incentive Spirometry (IS)

**Figure 1.**
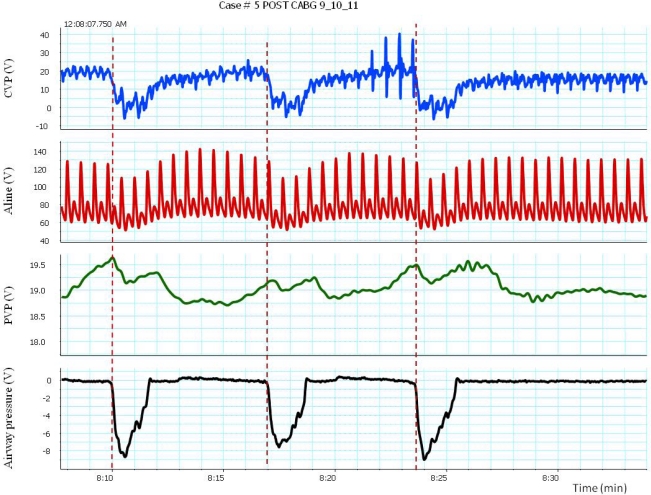
Impact of incentive spirometry, which induces a rapid deep breath, on arterial pressure, CVP and peripheral venous pressure (PVP). Notice that pulsus paradoxus occurs with inspiration.

**Figure 2.**
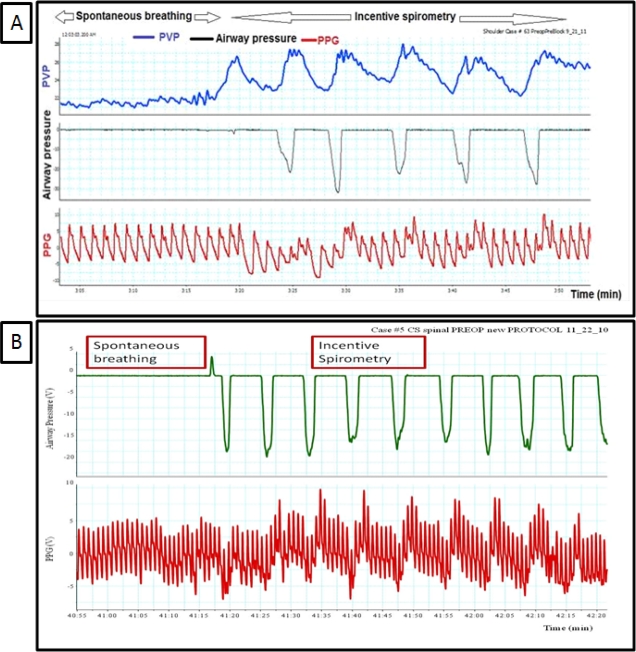
(**A**) and (**B**) show the impact of calm spontaneous breathing and incentive spirometry (deep rapid breathes) on peripheral venous pressure (PVP) and PPG waveforms, notice that incentive spirometry breathing is an exaggerated response of the spontaneous breathing effect.

Breathing through the incentive spirometry (IS) was associated with rapid expansion of the lung with a volume close to 2.5 to 3.5 liter with a simultaneous fall in Pa, PAP, and LAP and an increase in the peripheral venous pressure as shown in Figure 1. The impact of breathing through incentive spirometry is considered an exaggerated response of normal spontaneous breathing (Figure 2), as pleural pressure (Ppl) fell more than did chamber pressures, transmural LAP, PAP and aortic transmural pressure increased and that was associated with increased LV preload and afterload of both RV and LV (similar to spontaneous breathing). Rapid expansion of the lung will directly compress compliant vena cava, increases resistance to venous flow and results in reductions in venous return (reduction of RV preload) and an increase in the peripheral venous pressure (similar to controlled ventilation) as explained in Table 1.

**Figure 3.**
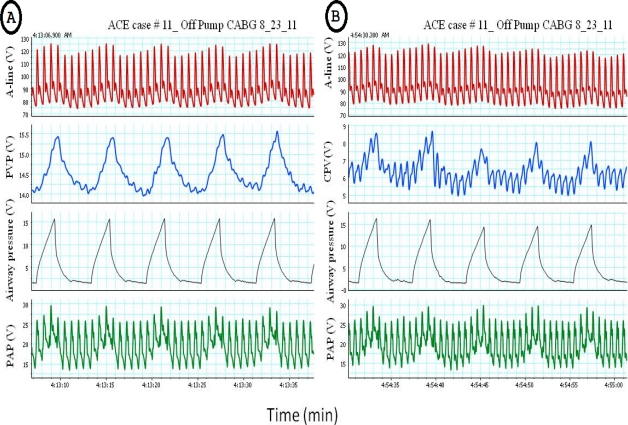
The impact of positive pressure ventilation on arterial pressure, pulmonary artery pressure (PAP), central venous pressure (CVP) and peripheral venous pressure (PVP), notice the transient increase in the arterial pressure(reverse pulsus paradoxus, RPP), PAP, CVP and PVP during the inspiratory cycle of controlled ventilation. Positive pressure ventilation will compress the vena cava and resulted in increased of both PVP (**A**) and CVP (**B**).

The transient increase in arterial pressure associated with positive pressure inspiration is termed reversed pulsus paradoxus (RPP). Positive pressure ventilation was associated with increased pleural pressure (Ppl) together with mean arterial pressure (Pa), right atrial pressure (RAP), left atrial pressure (LAP) and peripheral venous pressure as shown in Figure 3. It has been shown that with positive pressure ventilation there was more increase in pleural pressure (Ppl) than the rise in mean arterial pressure (Pa) left atrial pressure (LAP) which results in reduction of the transmural chamber pressure, which represents the actual filling pressures, of the aorta and LAP respectively. The decreased in transmural LAP could result from either diminished afterload of LV or decreased systemic venous return. Compression of the compliant vena cava and increases resistance to venous flow results in reductions in systemic venous return [13]. Thus, RPP occurred when LV preload and afterload, as estimated from transmural LAP and the transmural aortic pressure respectively, were actually decreased [21].

**Figure 4.**
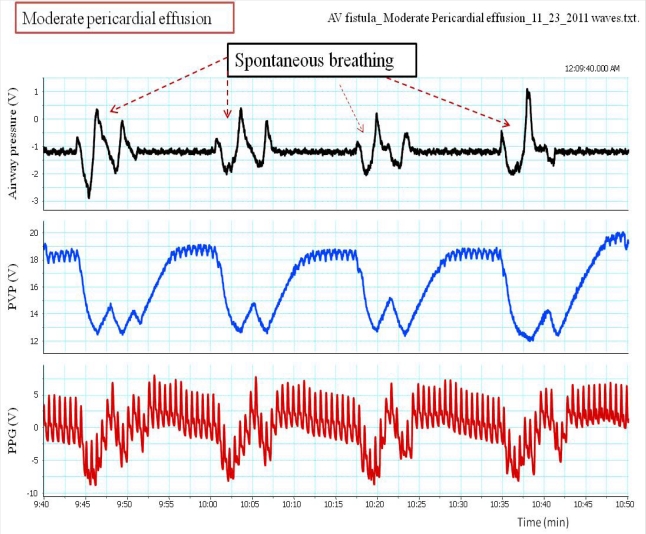
shows the impact of spontaneous breathing on the PPG and peripheral venous pressure (PVP), and airway pressure measured from a nasal cannula in the presence of a moderate pericardia effusion (early cardiac tamponade). Spontaneous breathing was associated with reduction in both PPG amplitude as well as peripheral venous pressure.

**Figure 5.**
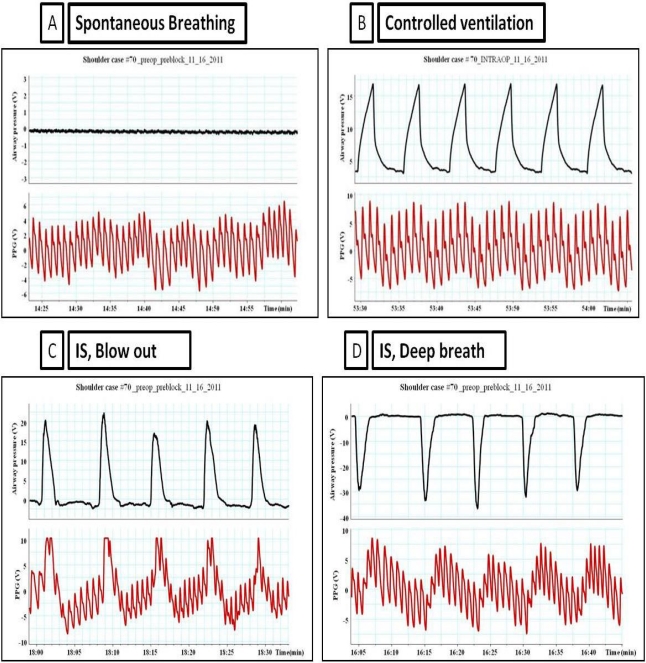
The impact of different types of ventilation on PPG waveforms. (**A**) Spontaneous breathing. (**B**) The impact of mechanical ventilation (volume controlled) on the PPG waveforms. (**C**) Patient is blowing out through the incentive spirometry; patient is generating positive 20 cm H_2_O, notice the change in PPG waveforms which looks like Valsalva maneuvers (looks like the same effect of expiratory resistor). (**D**) Patient taking deep breath through incentive spirometry, maximum negative airway pressure was a round 30 cm H_2_O, notice that PPG waveforms are an exaggeration of the normal spontaneous breathing.

**Pulsus paradoxus** is the pathological exaggeration of the normal transient decrease in arterial blood pressure during spontaneous breathing. During spontaneous breathing the pleural pressure decreased relative to atmospheric pressure with simultaneous fall in arterial pressure, LAP, PAP and RAP. Pleural pressure fell more than did chamber pressures which resulted in an increased in transmural RA, LA and aortic pressures. Spontaneous breathing was associated with augmentation of LA filling pressure as a result of increased transmural RAP which promotes systemic venous return and increased LV afterload. Despite this increase in LV filling pressure, systemic arterial pressure decreased due to increased LV afterload during spontaneous inspiration [21]. Comparison between pulsus paradoxus and reversed pulsus paradoxus is shown in Table 2. In certain pathological conditions such as cardiac tamponade, where the *left heart filling is hampered during inspiration,* or acute exacerbation of chronic obstructive lung disease or asthma when *the right heart filling is reduced during expiration* by high intrathoracic pressure, pulsus paradoxus may be enhanced [22,23]. Figure 4 shows the impact of spontaneous breathing on the PPG and peripheral venous pressure waveforms in patients with moderate pericardial effusion. Figures 5 through 13 given further examples of the interaction of the respiratory system and the PPG.

## 5. Conclusions

The use of the photoplethysmographic (PPG) waveform for patient monitoring offers a number of significant advantages. The technology has proven to be inexpensive, low risk, and easy-to-use by both the patient and the clinician. The primary goal for the clinical engineer should be to develop an understanding of the underlying physiology responsible for the waveform. Especially with the PPG where features that are originally considered to be artifactual in nature, have become a source of rich physiologic signal.

The next step is the utilization of this understanding to develop new methods of patient monitoring. It is important to keep in mind that monitors in themselves do not change patient outcomes, it is only change in therapy that can do that. The results of monitoring should be presented in such a way as to allow for guidance of therapy, which might take on many forms including pharmaceutical agents, the administration of intravenous fluids (saline or blood) or the administration of oxygen.

From the examples shown above it is obvious that ventilation is affecting the PPG waveforms morphology. Until recently, these changes in the waveforms have been seen as a source of artifact which interferes with the calculation of arterial saturation and further analysis of the waveforms. Modern digital signal processing is allowing for a re-examination of this ubiquitous waveform.

Evidence now suggests that the DC component (baseline modulation) is associated with the movement of venous blood [24,25]. Unlike static pre-load measurements (*i.e.*, CVP & PAP), which have a poor reputation for guiding fluid therapy, the potential of using respiratory induced peripheral venous modulation remains to be explored. From a theoretical view point, venous modulation should be immune to the effects of cardiac arrhythmia and vascular tone changes. In the long run, this approach may reveal a new method of monitoring cardiovascular physiology which would be focused on the pre-load side of the circulation.

There are already commercial medical devices (PVI, Masimo, Irving CA) on the market that measure arterial modulations (AC) which is felt to be due to modulations in stroke volume [26]. When used as an index of fluid responsiveness (e.g., predicting if a patients cardiac output would increase with a fluid bolus) this method of monitor has fared well [27]. The fact that information regarding both the pre-load condition and its effect on stroke volume is obtainable from a PPG is intriguing and may give insight in the underlying cardiac function.

## Figures and Tables

**Figure 6.**
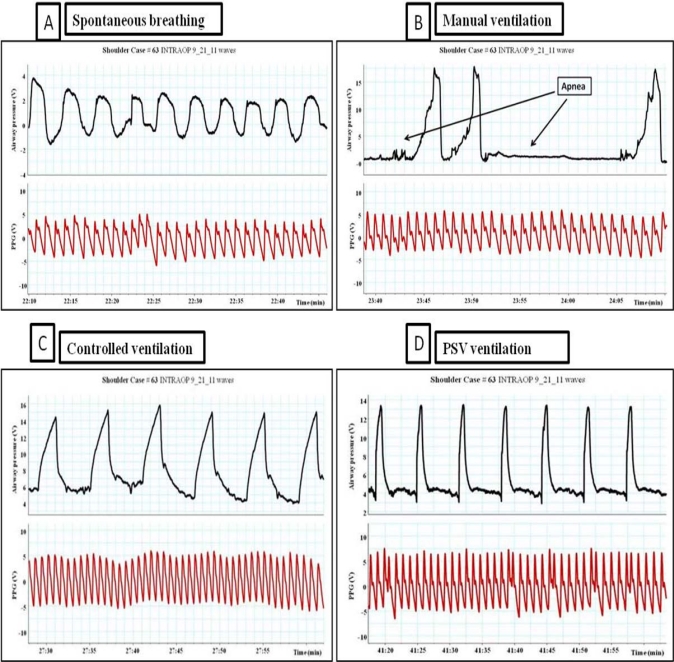
Impact of ventilation on PPG waveforms. (**A**) Spontaneous breathing. (**B**) Manual ventilation with periods of apnea. (**C**) Controlled mechanical ventilation (volume controlled ventilation), tidal volume = 500 cc. (**D**) The impact of PSV ventilation on the PPG waveforms, Peak pressure = 15 cmH_2_O, PEEP = 5 cmH_2_O, pressure support = 14 cmH_2_O, respiratory rate = 10, tidal volume = 300–350 cc.

**Figure 7.**
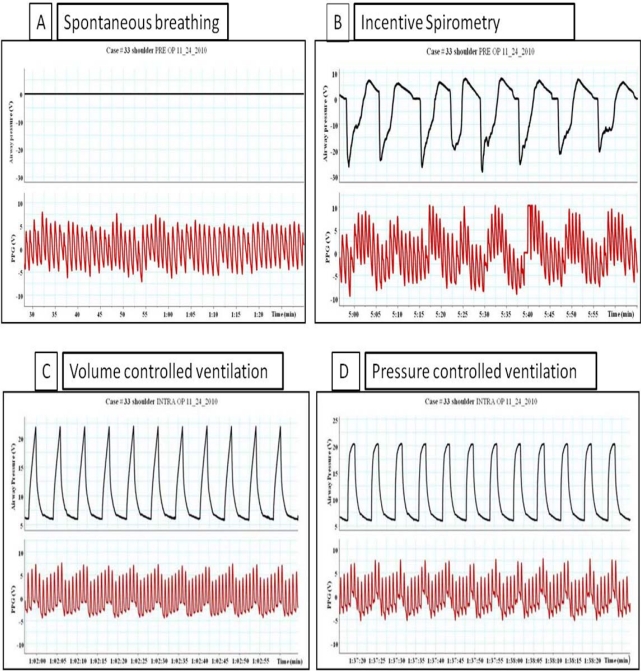
The impact of ventilation on PPG waveforms. (**A**) Spontaneous breathing, (**B**) Incentive spirometry breathing with negative airway pressure around 20 cm H_2_O. (**C**) Volume controlled mechanical ventilation (**D**) The impact of pressure controlled mechanical on the PPG waveforms.

**Figure 8.**
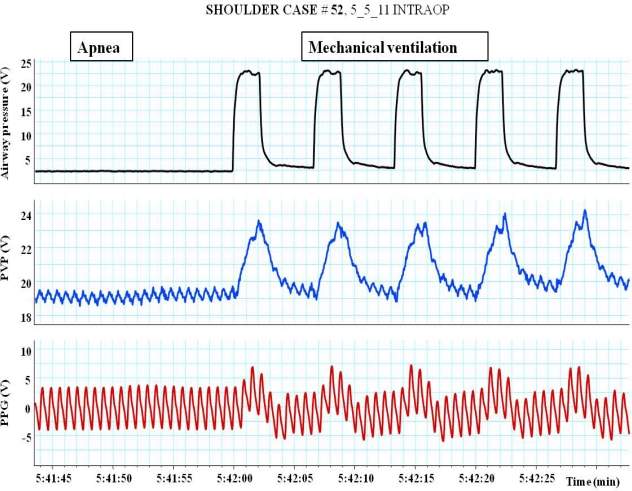
An example of the impact of pressure controlled ventilation (PCV) and apnea on peripheral venous pressure (PVP) and plethysmographic (PPG) waveforms. Each positive pressure ventilation was associated with an increased in peripheral venous pressure (PVP) and PPG amplitude as a result of right ventricle preload and left ventricular afterload reduction respectively.

**Figure 9.**
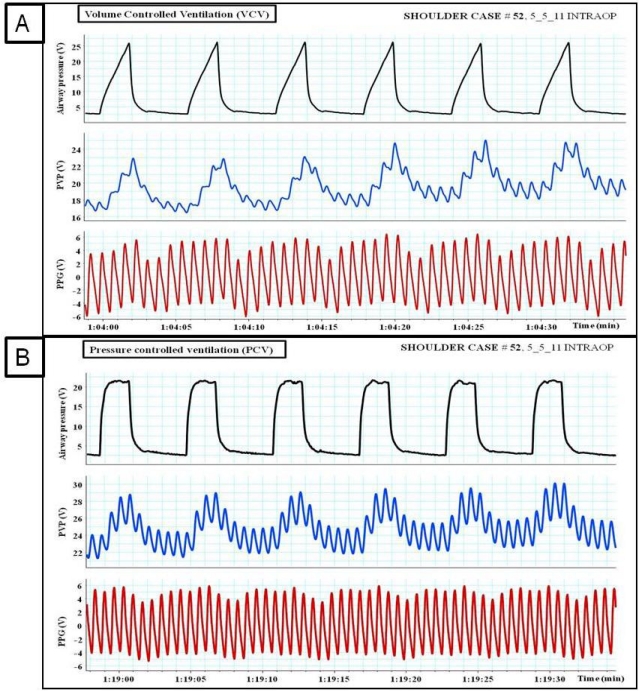
(**A**) The impact of volume controlled ventilation (VCV) and (**B**) pressure controlled ventilation (PCV) on peripheral venous pressure (PVP) and plethysmographic (PPG) waveforms.

**Figure 10.**
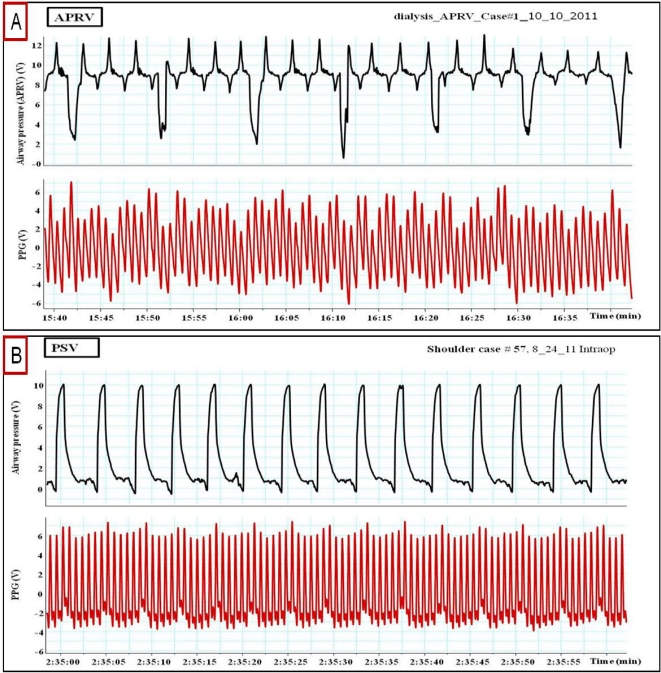
(**A**) Impact of Airway Pressure Release Ventilation (APRV) and (**B**) Pressure Support Ventilation (PSV pro) on peripheral venous pressure (PVP) and plethysmographic (PPG) waveforms.

**Figure 11.**
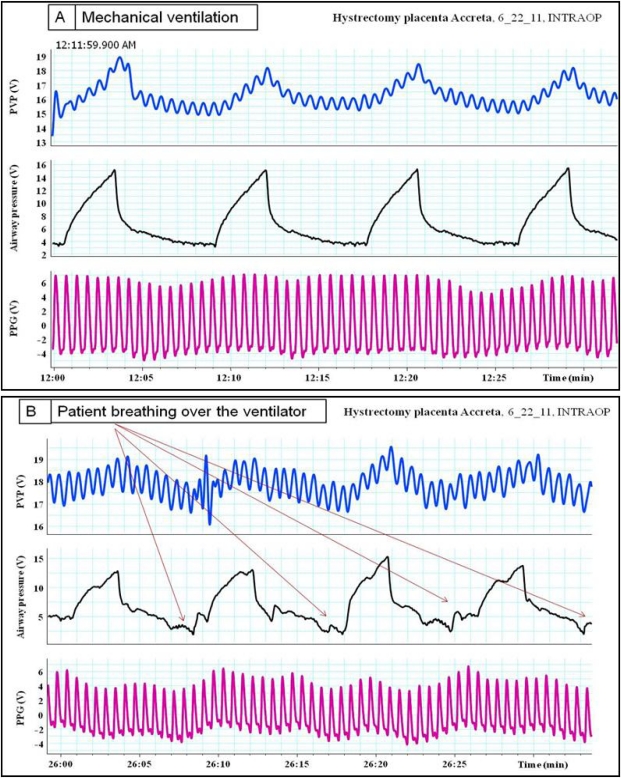
(**A**) Volume controlled mechanical ventilation. (**B**) The same patient when she started to breath over the ventilator. Notice the modulation of plethysmographic waveforms and changes in the pattern of peripheral venous pressure (PVP) waveforms.

**Figure 12.**
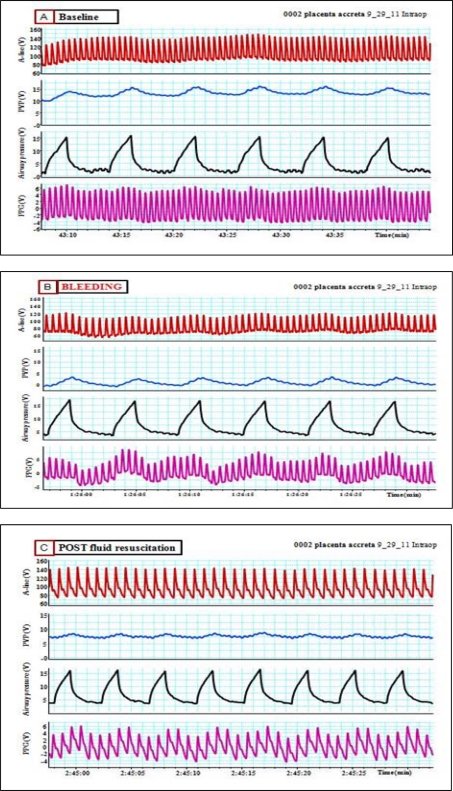
(**A**) (upper panel): **Baseline**; arterial blood pressure (A-line), peripheral venous pressure (PVP) and plethysmographic waveforms during volume controlled mechanical ventilation. (**B**) (middle panel): **Post bleeding**; notice the drop in the peripheral venous pressure together with the respiratory modulation of arterial blood pressure and PPG waveforms. (**C**) (lower panel): **Post fluid resuscitation**; shows partial restoration of peripheral venous pressure together with less oscillation of PPG and arterial blood pressure.

**Figure 13.**
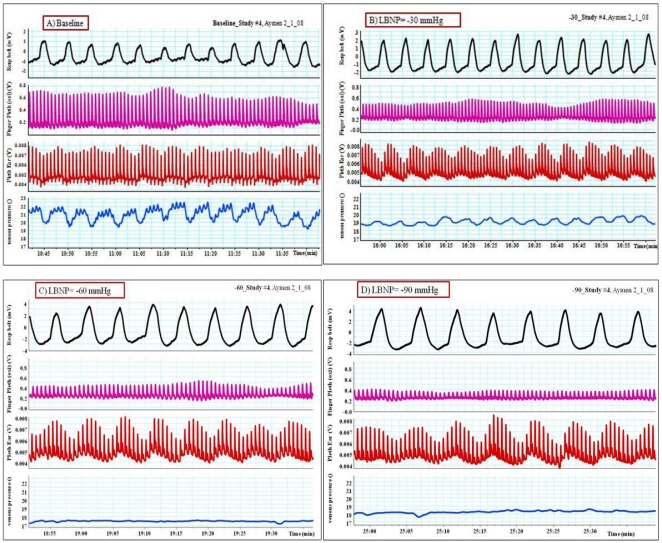
Changes in the plethysmographic waveforms from the ear and the finger together with changes in the peripheral venous pressure in spontaneously breathing patients underwent simulating hypovolemia by application of lower body negative pressure to the lower extremities. (**A**) Baseline, (**B**) LBNP = −30 mmHg, (**C**) LBNP = −60 mmHg and (**D**) LBNP = −90 mmHg. Notice the drop in the PVP and the marked vasoconstriction of finger PPG while the ear PPG showing an increase in the modulation with the progress of LBNP protocol.

**Table 1.** The impact of mechanical ventilation, spontaneous breathing and spontaneous breathing through the incentive spirometry on right and left ventricular preload and afterload. Bothe positive pressure inspiration and spontaneous inspiration increase pulmonary vascular resistance RV afterload).

**Mechanical ventilation**		
	Right Ventricle (RV)	Left Ventricle (LV)
Preload	Decreased	Decreased
Afterload	Increased	Decreased
**Spontaneous breathing**		
	Right Ventricle (RV)	Left Ventricle (LV)
Preload	Increased	Increased
Afterload	Increased	Increased
**Spontaneous breathing with incentive spirometry (IS)**		
	Right Ventricle (RV)	Left Ventricle (LV)
Preload	Decreased	Increased
Afterload	Increased	Increased

**Table 2.** Comparison between pulsus paradoxus and reversed pulsus paradoxus.

	Pulsus paradoxus Inspiration (spontaneous. Breathing)	Reverse Pulsus paradoxus (RPP) Inspiration (Positive pressure ventilation)
RA pressure	decreased	increased
Transmural RA, LA, aortic pressure	increased	decreased
LVESV	increased	decreased
LVEDV	No change	No change
LV afterload	**increased**	**decreased**
Arterial pressure	decreased	Transient Increased
